# A Measure of Uncertainty regarding the Interval Constraint of Normal Mean Elicited by Two Stages of a Prior Hierarchy

**DOI:** 10.1155/2014/676545

**Published:** 2014-07-06

**Authors:** Hea-Jung Kim

**Affiliations:** Department of Statistics, Dongguk University, Seoul Campus, Pil-Dong 3Ga, Chung-Gu, Seoul 100-715, Republic of Korea

## Abstract

This paper considers a *hierarchical screened Gaussian model* (HSGM) for Bayesian inference of normal models when an interval constraint in the mean parameter space needs to be incorporated in the modeling but when such a restriction is uncertain. An objective measure of the uncertainty, regarding the interval constraint, accounted for by using the HSGM is proposed for the Bayesian inference. For this purpose, we drive a maximum entropy prior of the normal mean, eliciting the uncertainty regarding the interval constraint, and then obtain the uncertainty measure by considering the relationship between the maximum entropy prior and the marginal prior of the normal mean in HSGM. Bayesian estimation procedure of HSGM is developed and two numerical illustrations pertaining to the properties of the uncertainty measure are provided.

## 1. Introduction

Consider the following model for normally distributed data:

(1) $$\begin{array}{c} y_{i}=\theta +\epsilon_{i},\;\epsilon_{i}\sim N\left(0,\tau^{2}\right),\;\;i=1,\ldots ,n. \end{array}$$

Bayesian analysis of the model (1) begins with the specification of prior distributions for unknown parameters *θ* and the noise variance *τ* ^2^. Specifically, we assign a normal prior distribution for *θ* and an inverse gamma (IG) prior for *τ* ^2^, that is, *θ* ~ *N*(*μ*, *σ* ^2^) and *τ* ^2^ ~ IG(*c*, *d*), which are commonly used in a normal model as conjugate priors, where all the hyperparameters *μ* and *σ* ^2^,  *c*, and *d* are assumed to be known in the first place.

On the other hand, when we are completely sure about a functional constraint of *θ* a priori; a suitable restriction on the parameter space Θ = *R*, such as using a truncated normal distribution, is expected. However, it is often the case that the actual observations of (1) may violate the constraint on account of the measurement error or due to some other reasons. Further, the data may provide strong evidence that the constraint is inappropriate and therefore may appear to contradict the theory associated with the constraint. In this respect, it is expected that the uncertainty about the constraint is taken into account in the estimation procedure. O'Hagan and Leonard [1] indeed proposed two stages of a prior hierarchy based on the truncated prior distribution, which reflects the uncertainty about the parameter constraint. Liseo and Loperfido [2], Kim [3], and Kim and Choi [4] among others considered the Bayesian estimation of normal models with uncertain interval constraints using the idea of two stages of a prior hierarchy. In particular, Kim [3] obtained the marginal prior of *θ* as the normal selection distribution (e.g., [5]) and thus exploited the class of weighted normal distribution by Kim [6] for reflecting the uncertain prior belief on *θ*.

Although the two-stage prior is applied by many investigators, there is no objective method to measure (or control) the degree of uncertainty regarding the interval constraint of *θ* accounted for by using the two stages of a prior hierarchy. This is a major hindrance factor in developing the idea of the two stages of a prior hierarchy which is advocated by O'Hagan and Leonard [1]. Thus, such practical problem motivates us to develop a formal measure of uncertainty about the constraint in order to show how the uncertainty of the prior information regarding interval constrained parameter space Θ of *θ* can be reflected by utilizing the two stages of a prior hierarchy. This topic is tackled in this paper.

To propose the uncertainty measure, we consider the Bayesian inference of the normal mean *θ* in (1), but subject to an uncertain interval constraint. Because the maximum entropy prior by Jaynes [7, 8] is useful for describing (or measuring) the relative levels of uncertainty about the distribution of the prior parameter, our investigation focuses on the theoretical relationship between the two stages of a prior hierarchy of *θ* by O'Hagan and Leonard [1] and the maximum entropy prior subject to an uncertain interval constraint. The remainder of this paper is organized as follows. In Section 2, we briefly discuss the two-stage prior of *θ*, which will be used for the Bayesian analysis of *θ* subject to uncertainty regarding the interval constraint. Accordingly, influenced by the seminal work of O'Hagan and Leonard [1], we provide a normal model based on the two-stage prior distribution of *θ*, referred to as* hierarchical screened Gaussian model (HSGM)*. In Section 3, we explore the theoretical properties of the two-stage prior of *θ* by using Boltzmann's maximum entropy theorem [9, 10]. Based on the properties, we propose an objective measure of uncertainty regarding the interval constraint of *θ* that is accounted for by the two-stage prior. In Section 4, we explore Bayesian estimation procedure by analytically deriving the posterior distribution of the unknown parameters under HSGM, and we discuss the properties of the proposed measure of uncertainty that can be explained in the context of HSGM. Finally, the concluding remarks along with a discussion are made in Section 5.

## 2. Hierarchical Screened Gaussian Model

Let us assume the normal model (1) and consider the two stages of a prior hierarchy in the following way:

(2) yi=θ+ϵi, ϵi~N(0,τ2),  i=1,…,n,τ2~IG(c,d),θ ∣ θ0~N(θ0,σ02), independent  of  (ϵ1,…,ϵn)⊤,θ0~N(a≤θ0≤b)(μ,σ12),

where *N*_(*a*≤*μ*≤*b*)_(*μ*, *σ*_1_^2^) denotes a doubly truncated *N*(*μ*, *σ*_1_^2^) distribution with the lower truncation point *a* and upper truncation point *b*. In practice, there are certain cases in which we have a priori information that *θ* is highly likely to have an interval constraint, and thus the value of *θ* needs to be located with uncertainty in a restricted space Θ,

(3) $$\begin{array}{c} \Theta =\left\{\theta ;a\leq \theta \leq b\right\}\;\text{or},\;\;\text{simply},\;\;\;\Theta =\left[a,b\right]. \end{array}$$

In order to elicit the prior distribution on the uncertain interval constraint, we utilize the two-stage hierarchical model as in (2), which was initially advocated by O'Hagan and Leonard [1] in which values of *θ* that do not belong to Θ are penalized to a lesser extent. In this respect, the normal model structure of (2) is referred to as HSGM in the remainder of the paper, because the two stages of a prior hierarchy on *θ* are considered and the resulting marginal prior distribution of *θ* becomes the weighted normal (or interval screened normal) distribution studied by Kim [6]. This is shown as follows. Since *θ*_0_ ~ *N*_(*a*,*b*)_(*μ*, *σ*_1_^2^), the marginal prior of *θ* under HSGM is

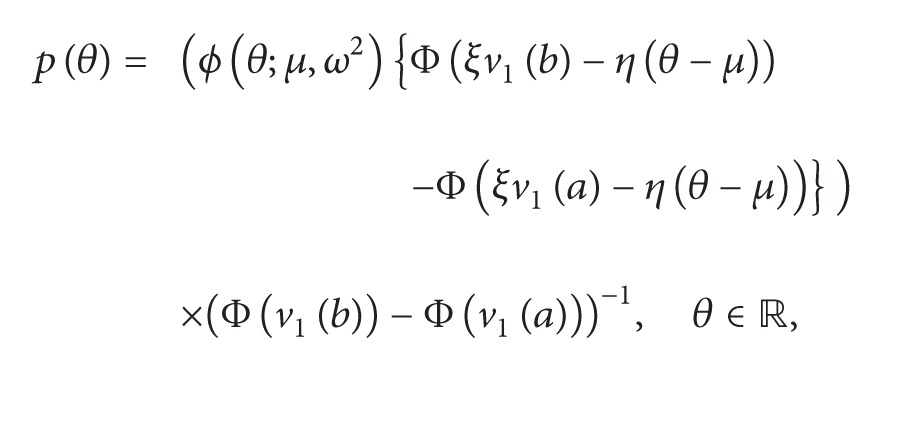
(4)
where *ϕ*(·; *μ*, *w* ^2^) and Φ(·), respectively, denote the density of *N*(*μ*, *w* ^2^) and the distribution function of *N*(0,1), *ω* ^2^ = *σ*_0_^2^ + *σ*_1_^2^, *v*_1_(*a*) = (*a* − *μ*)/*σ*_1_, *v*_1_(*b*) = (*b* − *μ*)/*σ*_1_, *ξ* = *ω*/*σ*_0_ and *η* = *ξσ*_1_/*ω* ^2^.

We see that (4) is the density of a weighted normal (WN) distribution by Kim [6]. This leads to the following assertion in Lemma 1.

**Lemma 1 .**

The two-stage prior of *θ* in HSGM of (2), eliciting the uncertain interval constraint (3), is marginally distributed as a weighted normal distribution,

(5) $$\begin{array}{c} \theta \sim WN_{\left(a,b\right)}\left(\mu ,\Omega \right) \end{array}$$

with its density (4), where

(6) $$\begin{array}{c} \mu =\left(\begin{array}{c} \mu \\ \mu \end{array}\right),\;\;\Omega =\left(\begin{array}{cc} \sigma_{1}^{2} & \sigma_{1}^{2}\\ \sigma_{1}^{2} & \sigma_{0}^{2}+\sigma_{1}^{2} \end{array}\right). \end{array}$$

Note that $\theta \overset{d}{=}[V|a\leq U\leq b]$, where (*U*, *V*)^*⊤*^ ~ *N*_2_(***μ***, Ω), a bivariate normal distribution. See Kim [6] for various properties of the WN_(*a*,*b*)_(***μ***, Ω) distribution. According to O'Hagan and Leonald [1], the first stage variance, *σ*_0_^2^, of the mean may measure the degree of uncertainty in the constraint. However, there is no systematic method to assign the values of *σ*_0_^2^ (or *σ*_1_^2^), according to a priori specified degree (say, (1 − *α*) × 100%) of uncertainty about the interval constraint (3). This is a major hindrance factor in developing the HSGM.

## 3. The Measure of Uncertainty

### 3.1. A Maximum Entropy Prior

Sometimes we have a situation where partial prior information is available, outside of which it is desired to use a priori that is as noninformative as possible. A useful method of dealing with this problem is through the concept of entropy by Jaynes [7, 8]. As discussed by Rosenkrantz [11], entropy has a direct relationship to information theory and in a sense measures the amount of uncertainty inherent in the probability distribution.

Assume now that we can specify the partial information concerning a location parameter *θ* (including the normal mean) with continuous space Θ of the form

(7) $$\begin{array}{c} E\left[t_{j}\left(\theta \right)\right]=\int_{\Theta }t_{j}\left(\theta \right)\pi \left(\theta \right)d\theta =t_{j},\;j=1,\ldots ,k, \end{array}$$

but with nothing else about our prior distribution *π*(*θ*). Then the maximum entropy prior can be obtained by choosing *π*(*θ*) that maximizes the entropy

(8) $$\begin{array}{c} \xi \left(\pi \right)=-\int_{\Theta }\pi \left(\theta \right)\log\pi \left(\theta \right)d\theta , \end{array}$$

in the presence of the partial information in the form of (7). A straightforward application of the calculus of variation leads us to Boltzmann's maximum entropy theorem. This tells us that the density *π*(*θ*) that maximizes *ξ*(*π*), subject to the constraints *E*[*t*_*j*_(*θ*)] = *t*_*j*_, *j* = 1,…, *k*, takes the *k*-parameter exponential family form

(9) $$\begin{array}{c} \pi \left(\theta \right)\propto \exp\left\{\lambda_{1}t_{1}\left(\theta \right)+\lambda_{2}t_{2}\left(\theta \right)+\cdots +\lambda_{k}t_{k}\left(\theta \right)\right\},\;\theta \in \Theta , \end{array}$$

where *λ*_1_, *λ*_2_,…, *λ*_*k*_ can be determined, via the *k*-constraints, in terms of *t*_1_,…, *t*_*k*_. See Leonard and Hsu [12] for the proof.

### 3.2. Maximum Entropy Prior for Constrained Normal Mean

Let the location parameter *θ* of our interest be the normal mean in (1). Then the results of the previous subsection can be applied to the prior for the normal mean *θ*. This subsection considers the case where the partial priori information of *θ* is in the form of an interval, that is, *θ* ∈ Θ, Θ = [*a*, *b*] with *a* < *b*, and examines how the maximum entropy prior of *θ*, that is, *π*(*θ*), has different formula according to the degree of uncertainty regarding the interval constraint.

**Case 1 .**

Θ = *R*, and *θ* ∈ Θ with certainty.

When we have partial priori information about *θ* that we can specify values for both mean *μ* and variance *σ* ^2^. Then the *N*(*μ*, *σ* ^2^) prior specification is the maximum entropy prior for *θ* (e.g., [12]). Thus the prior density and its entropy for the Case 1 are

(10) π1(θ)=ϕ(θ;μ,σ2), θ∈R,Ent(π1(θ))=−∫Θϕ(θ;μ,σ2)ln⁡ϕ(θ;μ,σ2)dθ=12(1+ln⁡(2π)+ln⁡σ2),

respectively.

**Case 2 .**

Θ = [*a*, *b*], and *θ* ∈ Θ with certainty.

On the other hand, when we are completely sure about the priori interval constraint of *θ*, a suitable restriction on the parameter space Θ such as using a truncated distribution is expected. This case supposes that we can specify values for both *E*[*θ*] = *μ* and *E*[(*θ* − *μ*)^2^] = *σ* ^2^ on the space *θ* ∈ *R* by a priori information. Further suppose that we have certain prior information that the parameter *θ* is concentrated on the region [*a*, *b*], that is,

(11) $$\begin{array}{c} p\left(a\leq \theta \leq b\right)=1 \end{array}$$

but nothing else about our prior distribution *π*(*θ*). The last condition is equivalent to *E*[*I*(*a* ≤ *θ* ≤ *b*)] = 1. Therefore, by Bolzmann's maximum entropy theorem, the prior density of *θ* for the Case 2 is

(12) π2(θ)∝exp⁡{λ1t1(θ)+λ2t2(θ)+λ3t3(θ)}∝exp⁡⁡{[θ−(μ−λ1/2λ2)]2λ2−1} ×exp⁡⁡{λ3I(a≤θ≤b)}

by (9), because *t*_1_(*θ*) = *θ*, *t*_1_ = *μ*, *t*_2_(*θ*) = (*θ* − *μ*)^2^, *t*_2_ = *σ* ^2^, and *t*_3_(*θ*) = *I*  (*a* ≤ *θ* ≤ *b*). Since *t*_3_ = 1, the maximum entropy prior, subject to these three restrictions, is thus

(13) $$\begin{array}{c} \pi_{2}\left(\theta \right)=\frac{\phi \left(\theta ;\mu ,\sigma^{2}\right)}{\Phi \left(v\left(b\right)\right)-\Phi \left(v\left(a\right)\right)},\;a\leq \theta \leq b, \end{array}$$

provided that we choose *λ*_1_ = 0, *λ*_2_ = −1/2*σ* ^2^, *λ*_3_ = −ln⁡{Φ(*v*(*b*)) − Φ(*v*(*a*))}, where *v*(*a*) = (*a* − *μ*)/*σ* and *v*(*b*) = (*b* − *μ*)/*σ*.

Some algebra using the moments of the *N*_(*a*,*b*)_(*μ*, *σ* ^2^) distribution in Johnson et al. [13] yields the entropy of *π*_2_(*θ*) which is

(14) Ent(π2(θ)) =12(1+ln⁡⁡(2πσ2)+v(a)ϕ(v(a))−v(b)ϕ(v(b))Φ(v(b))−Φ(v(a)))  +ln⁡(Φ(v(b))−Φ(v(a))).

**Case 3 .**

Θ = [*a*, *b*], and *θ* ∈ Θ with (1 − *α*) × 100% uncertain.

Now suppose that we have partial priori information that we can specify values for both mean *μ* and variance *σ* ^2^ of *θ* for *θ* ∈ *R*. In addition, suppose that we have priori uncertain interval constraint information that *θ* ∈ [*a*, *b*]. Then along with the priori moment conditions *E*[*θ*] = *μ* and *E*[(*θ* − *μ*)^2^] = *σ* ^2^, the additional uncertain prior information about the interval constraint can be expressed by

(15) $$\begin{array}{c} p\left(a\leq \theta \leq b\right)=\alpha ,\;0\leq \alpha \leq 1, \end{array}$$

where 1 − *α* (or (1 − *α*) × 100%) denotes the degree of uncertainty. Thus the priori uncertain interval constraint is equivalent to

(16) $$\begin{array}{c} E\left[I\left(a\leq \theta \leq b\right)\right]=\alpha . \end{array}$$

Applying this partial prior information to Bolzmann's maximum entropy theorem, we have the following lemma.

**Lemma 2 .**

If $\alpha ={\bar{\Phi }}_{2}((a,b);\mu ,\Sigma )/(\Phi (v^{*}(b))-\Phi (v^{*}(a)))$, the maximum entropy prior distribution of *θ*, reflecting the uncertain interval constraint in (15) is

(17) $$\begin{array}{c} \theta \sim WN_{\left(a,b\right)}\left(\mu ,\Sigma \right), \end{array}$$

where $v^{*}(a)=(a-\mu )/(\sigma \sqrt{\delta })$, $v^{*}(b)=(b-\mu )/(\sigma \sqrt{\delta })$, $a={\left(a,a\right)}^{⊤},b={\left(b,b\right)}^{⊤},{\bar{\Phi }}_{2}((a,b):\mu ,\Sigma )$ denote a rectangle probability *p*(*a* ≤ *X*_2_ ≤ *b*, *a* ≤ *X*_1_ ≤ *b*) of *X*_1_ and *X*_2_ whose joint distribution is a bivariate normal *N*_2_(***μ***, Σ),

(18) $$\begin{array}{c} \mu =\left(\begin{array}{c} \mu \\ \mu \end{array}\right),\;\;\;\Sigma =\sigma^{2}\left(\begin{array}{cc} \delta & \delta \\ \delta & 1 \end{array}\right),\;0<\delta <1. \end{array}$$

**Proof**

Taking *t*_1_(*θ*) = *θ*, *t*_1_ = *μ*, *t*_2_(*θ*) = (*θ* − *μ*)^2^, *t*_2_ = *σ* ^2^, *t*_3_(*θ*) = *I*  (*a* ≤ *θ* ≤ *b*), and *t*_3_ = *α*, and setting *λ*_1_ = 0 and *λ*_2_ = −1/2*σ* ^2^, the maximum entropy prior *π*(*θ*) in (9) reduces to

(19) π3(θ)∝exp⁡{(θ−μ)22σ2}exp⁡{λ3t3(θ)}, θ∈R,

by Bolzmann's maximum entropy theorem. Now the second exponential term in the right hand side of (19) can be determined by using the condition (16) as follows. Among all the possible proper prior densities of the form (19), the choice of

(20) λ3t3(θ) =ln⁡{Φ(ξ∗v∗(b)−η∗(θ−μ))−Φ(ξ∗v∗(a)−η∗(θ−μ))Φ(v∗(b))−Φ(v∗(a))}

yields the proper prior density (22).  Further, this choice leads to the only proper prior that satisfies

(21) α=p(a≤X2≤b ∣ a≤X1≤b)    =Φ¯2((a,b);μ,Σ)(Φ(v∗(b))−Φ(v∗(a)))

in the condition (16), because (22) is the density of $\theta \overset{d}{=}[X_{2}|a\leq X_{1}\leq b]\sim \text{W}{\text{N}}_{\left(a,b\right)}(\mu ,\Sigma )$.

Thus the maximum entropy prior density for the Case 3 is given by

(22) π3(θ) =ϕ(θ;μ,σ2)  ×Φ(ξ∗v∗(b)−η∗(θ−μ))−Φ(ξ∗v∗(a)−η∗(θ−μ))Φ(v∗(b))−Φ(v∗(a)), θ∈R,

where ξ* = (1 − *δ*)^−1/2^ and $\eta^{*}=\xi^{*}\sqrt{\delta }/\sigma$.

Note, from Lemma 2, that *π*_1_(*θ*) = *π*_2_(*θ*) = *π*_3_(*θ*) for *a* = −*∞* and *b* = *∞*. This is consistent with our partial priori information that *E*[*θ*] = *μ* and Var⁡(*θ*) = *σ* ^2^ for *θ* ∈ *R*. Some algebra using the moments of the WN_(*a*,*b*)_(***μ***, Σ) distribution given in Kim [6] yields the entropy of *π*_3_(*θ*) (i.e., Ent(*π*_3_(*θ*))) given by

(23) 12{1+ln⁡(2πσ2)+v∗(a)ϕ(v∗(a))−v∗(b)ϕ(v∗(b))Φ(v∗(b))−Φ(v∗(a))δ} +ln⁡(Φ(v∗(b))−Φ(v∗(a)))   −∫Rln⁡{Φ(ξ∗v∗(b)−η∗(θ−μ)) −Φ(ξ∗v∗(a)−η∗(θ−μ))}π3(θ)dθ.

As seen in (23), the calculation of Ent(*π*_3_(*θ*)) involves a complicated integration. Instead, by using a Monte Carlo integration, we may calculate it approximately. According to Kim [6], it follows that the stochastic representation of the prior distribution *θ* ~ WN_(*a*,*b*)_(***μ***, Σ) with density *π*_3_(*θ*) is

(24) $$\begin{array}{c} \theta \;\overset{d}{=}\;\mu +\sigma \sqrt{\delta }\;Z_{\left(v^{*}(a),v^{*}(b)\right)}+\sigma {\left(1-\delta \right)}^{1/2}\;Z, \end{array}$$

where *Z*_(v*(*a*),v*(*b*))_ ~ *N*_(v*(*a*),v*(*b*))_(0,1) and *Z* ~ *N*(0,1), and they are independent. This stochastic representation is useful for generating *θ*'s from the prior distribution *θ*, and hence implementing the Monte Carlo integration. The following corollary asserts the relationship among *π*_*j*_(*θ*), *j* = 1,2, 3, in terms of *δ* ∈ (0,1).

**Corollary 3 .**

As *δ* → 1, the maximum entropy prior *π*_3_(*θ*) approximates to *π*_2_(*θ*) in (13), while *π*_3_(*θ*) is equivalent to *π*_1_(*θ*) for *δ* = 0.

**Proof**

Note from (22) that ξv*(*b*) − *η*(*θ* − *μ*) → *∞* and ξv*(*a*) − *η*(*θ* − *μ*)→−*∞* as *δ* → 1 for *θ* ∈ [*a*, *b*]. Also note, from the conditional property of *N*_2_(***μ***, Σ), that $\alpha ={\bar{\Phi }}_{2}((a,b);\mu ,\Sigma )/(\Phi (v^{*}(b))-\Phi (v^{*}(a)))\to 1$ is equivalent to *δ* → 1 for finite vales of *μ*,*σ*,*a*, and *b* with *b* > *a*. Applying these two results to *π*_3_(*θ*), we see that it approximates to *π*_2_(*θ*) as *δ* → 1. It is straightforward to see from Lemma 2 that  *π*_3_(*θ*) = *π*_1_(*θ*)  for *δ* = 0, because the WN_(*a*,*b*)_(***μ***, Σ) distribution is equivalent to *N*(*μ*, *σ* ^2^) for *δ* = 0.

**Figure 1:**
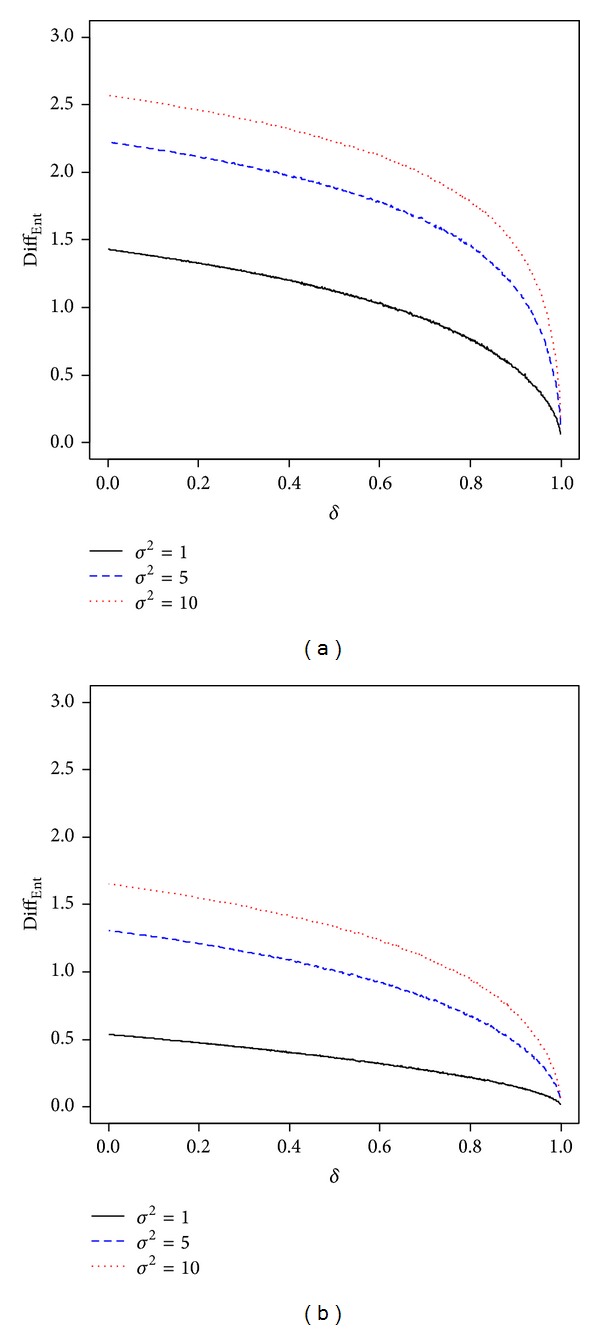
Plots of Diff_Ent_ = Ent(*π*_3_(*θ*)) − Ent(*π*_2_(*θ*)) for different values of *δ* ∈ (0,1) and *σ* ^2^ for the cases where (a) Θ = [−1.0,0] and (b) Θ = [−1.0,1.5].

Each graph of Figure 1 depicts the difference between Ent(*π*_3_(*θ*)) and Ent(*π*_2_(*θ*)) as a function of *δ* ∈ (0,1) for three values of *σ* ^2^, two cases of Θ = [*a*, *b*], and *μ* = 0. In Figure 1, the difference is denoted by Diff_Ent_. Since each graph coincides with the results of Corollary 3, we can obtain the following implications from the figure. (i) As expected, we see that Ent(*π*_1_(*θ*)) > Ent(*π*_3_(*θ*)) > Ent(*π*_2_(*θ*)) for *δ* ∈ (0,1). (ii) The entropy of *π*_3_(*θ*) is a monotone decreasing function of *δ*. (iii) Each entropy of the three priors increases as *σ* ^2^ becomes large. (iv) Diff_Ent_ is closely related with degree of uncertainty (i.e,. (1 − *α*) × 100%) for it is a monotone decreasing function of *δ* ∈ (0,1) and *α* is a function of *δ*. (v) Diff_Ent_ is a monotone increasing function of *σ* ^2^ for the case where a value of *δ* is given.

### 3.3. Objective Measure of Uncertainty

From a relationship between Lemmas 1 and 2, we can propose an objective measure for the degree of uncertainty regarding a prior interval constraint accounted for by HSGM. The following theorem proposes the objective measure using the same notations as used in Lemmas 1 and 2.

**Theorem 4 .**

Let *σ*_0_^2^ = (1 − *δ*)*σ* ^2^, *σ*_1_^2^ = *δσ* ^2^, and *δ* ∈ (0,1). Then the two-stage prior of *θ* defined by *HSGM* in (2) is equivalent to *π*_3_(*θ*) and the degree of uncertainty regarding the interval constraint, *θ* ∈ [*a*, *b*], accounted for by the *HSGM* is

(25) $$\begin{array}{c} \left\{1-\frac{{\bar{\Phi }}_{2}\left(\left(a,b\right);\mu ,\Sigma \right)}{\left(\Phi \left(v^{*}\left(b\right)\right)-\Phi \left(v^{*}\left(a\right)\right)\right)}\;\right\}\times 100\%. \end{array}$$

**Proof**

If *σ*_0_^2^ = (1 − *δ*)*σ* ^2^, *σ*_1_^2^ = *δσ* ^2^, and *δ* ∈ (0,1), the marginal prior distribution of *θ* in Lemma 1 is equivalent to the density of  WN_(*a*,*b*)_(***μ***, Σ). Thus the prior density *p*(*θ*) in (4) is equal to the maximum entropy prior *π*_3_(*θ*) in (22). Now Lemma 2 asserts that *π*_3_(*θ*) reflects uncertainty about the interval constraint *θ* ∈ [*a*, *b*] by the degree of (1 − *α*) × 100% with $\alpha ={\bar{\Phi }}_{2}((a,b);\mu ,\Sigma )/(\Phi (v^{*}(b))-\Phi (v^{*}(a)))$.

**Figure 2:**
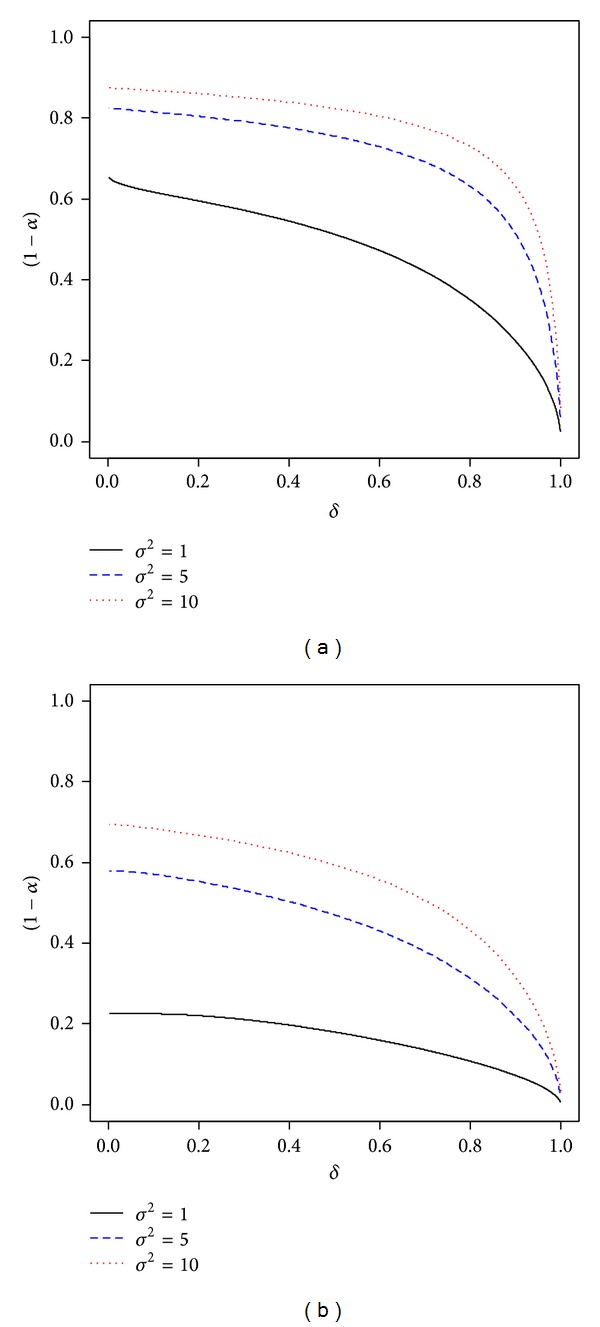
Plots of (1 − *α*) for different values of *δ* ∈ (0,1) and *σ* ^2^ for the cases where (a) Θ = [−1.0,0] and (b) Θ = [−1.0,1.5].

Theorem 4 provides an exact measure of the uncertainty about the priori interval constraint on *θ* accounted for by HSGM, and it shows that the uncertainty measure is different from that of O'Hagan and Leonald [1] which mainly depends on the first stage variance, *σ*_0_^2^, of *θ* in HSGM. Theorem 4 also indicates that HSGM in (2) can be used to elicit the priori uncertain interval information associated with *π*_3_(*θ*). Further, the entropy of the two-stage prior *θ* defined by the HSGM (i.e., *p*(*θ*) in (4)) can be calculated by using the formula of Ent(*π*_3_(*θ*)) in (23). We can visualize the degree of uncertainty about the priori interval constraint, *θ* ∈ [*a*, *b*], by plotting 1 − *α* for different values of *δ* ∈ (0,1) in Figure 2.

From Figure 2, we can see, for fixed value of *σ* ^2^, that HSGM with small values of *δ* tends to increase the uncertainty regarding the priori constraint. This coincides with the result which is asserted by Corollary 3. Further, we see from the figure that, for a fixed value of *δ* ∈ (0,1), we can enlarge (or reduce) the uncertainty about the priori constraint by increasing (or decreasing) the amount of *σ* ^2^ (or equivalently *σ*_0_^2^ and *σ*_1_^2^) in the two stages of a prior hierarchy of *θ*.

When the data information is *y* ~ *N*(*θ*, *τ* ^2^), it is well known that the maximum entropy priors (4) and (9) are conjugate priors for the normal mean *θ* when *τ* ^2^ is known. This is obtained from the following posteriors:

(26) p1(θ ∣ y)∝ϕ(y;θ,τ2)π1(θ)∝ϕ(θ;μ∗,σ∗2)for  θ∈R,p2(θ ∣ y)∝ϕ(y;θ,τ2)π2(θ)∝ϕ(θ;μ∗,σ∗2)  for  θ∈[a,b],

where μ* = (*σ* ^2^ *y* + *τ* ^2^ *μ*)/(*σ* ^2^ + *τ* ^2^) and *σ* ^∗2^ = *σ* ^2^ *τ* ^2^/(*σ* ^2^ + *τ* ^2^). Thus, we see that each prior satisfies the conjugate property that *π*_*j*_(*θ*) and *p*_*j*_(*θ*∣*y*) belong to the same family of distributions for *j* = 1,2. The following corollary provides this conjugate property which also applies to *π*_3_(*θ*) in (22).

**Corollary 5 .**

Let *y* ~ *N*(*θ*, *τ* ^2^) with known *τ* ^2^. Then the prior *π*_3_(*θ*) yields the posterior distribution of *θ* given by

(27) $$\begin{array}{c} \left(\theta \;|\;y\right)\sim WN_{\left(a,b\right)}\left(\mu^{*},\Sigma^{*}\right), \end{array}$$

where

(28) μ∗=((1−δ)μ+δμ∗μ∗),Σ∗=(δ(1−δ)σ2+δ2σ∗2δσ∗2δσ∗2σ∗2),

μ* = (*σ* ^2^ *y* + *τ* ^2^ *μ*)/(*σ* ^2^ + *τ* ^2^), and *σ* ^∗2^ = *σ* ^2^ *τ* ^2^/(*σ* ^2^ + *τ* ^2^).

**Proof**

Since *y* ~ *N*(*θ*, *τ* ^2^) with known *τ* ^2^, under the prior *π*_3_(*θ*) in (22), the posterior density of *θ* is given by

(29) p(θ ∣ y)∝ϕ(y;θ,τ2)ϕ(θ;μ,σ2)  ×{Φ(ξ∗v∗(b)−η∗(θ−μ))−Φ(ξ∗v∗(a)−η∗(θ−μ))}  ∝ϕ(θ;μ∗,σ∗2){Φ(ξ∗γ(b)−η∗(θ−μ∗)) −Φ(ξ∗γ(a)−η∗(θ−μ∗))}

in that {Φ(ξ*v*(*b*) − η*(*θ* − *μ*)) − Φ(ξ*v*(*a*) − η*(*θ* − *μ*))} = {Φ(ξ*γ(*b*) − η*(*θ* − μ*)) − Φ(ξ*γ(*a*) − η*(*θ* − μ*))}, where $\gamma (b)=\{b-((1-\delta )\mu +\delta \mu^{*})\}/(\sigma \sqrt{\delta })$ and $\gamma (a)=\{a-((1-\delta )\mu +\delta \mu^{*})\}/(\sigma \sqrt{\delta })$. This is a kernel of WN_(*a*,*b*)_(μ*, Σ*) density.

## 4. Posterior Estimation

Let us revisit the HSGM with the following two stages of a prior hierarchy of *θ*:

(30) $$\begin{array}{c} y_{i}=\theta +\epsilon_{i},\;\epsilon_{i}\sim N\left(0,\tau^{2}\right),\;\;i=1,\ldots ,n,\\ \tau^{2}\sim \text{IG}\left(c,d\right),\\ \theta \;|\;\theta_{0}\sim N\left(\theta_{0},\left(1-\delta \right)\sigma^{2}\right),\;\text{independent}\;\text{of}\;{\left(\epsilon_{1},\ldots ,\epsilon_{n}\right)}^{⊤},\\ \theta_{0}\sim N_{\left(a\leq \theta_{0}\leq b\right)}\left(\mu ,\delta \sigma^{2}\right),\;\delta \in \left(0,1\right). \end{array}$$

According to Theorem 4, we see that the two-stage prior of *θ* is essentially the same as the maximum entropy prior which properly elicits the partial priori information of an uncertain interval constraint, {*θ*; *θ* ∈ [*a*, *b*]}, with (1 − *α*) × 100% degree of uncertainty, where $\alpha ={\bar{\Phi }}_{2}((a,b);\mu ,\Sigma )/(\Phi (v^{*}(b))-\Phi (v^{*}(a)))$. Here *μ* and *σ* ^2^ are true prior mean and variance of the parameter *θ* when the uncertain priori interval condition does not exist.

Based on the marginal prior distribution of *θ* in Lemma 1, we have the joint posterior distribution of *θ* and *τ* ^2^ proportional to the product of likelihood and the prior distribution,

(31) $$\begin{array}{c} p\left(\theta ,\tau^{2}\;|\;D_{n}\right)\propto \overset{n}{\underset{i=1}{\prod }}\phi \left(y_{i};\theta ,\tau^{2}\right)\pi_{3}\left(\theta \right)\text{IG}\left(\tau^{2};c,d\right), \end{array}$$

where *D*_*n*_ = {*y*_1_,…, *y*_*n*_}, IG(·; *c*, *d*) is the inverse-gamma density with parameters *c* and *d*, and *π*_3_(*θ*) is the density (22), that is, the density of *θ* ~ WN_(*a*,*b*)_(***μ***, Σ). Note that the joint posterior is not simplified in an analytic form of the known density and thus intractable for posterior inference. Instead, we derive each of the conditional posterior distributions of *θ* and *τ* ^2^ in an explicit form, which will be useful for posterior inference such as Gibbs sampling (e.g., [14]).

**Corollary 6 .**

Given the joint posterior distribution (31), we have the following.

(i) The full conditional posterior distribution of *θ* is given by
  (32) $$\begin{array}{c} \theta \;|\;\left(\tau^{2},D_{n}\right)\sim WN_{\left(a,b\right)}\left(\theta^{*},\Omega^{*}\right), \end{array}$$

where

(33) θ∗=((1−δ)μ+δθ∗θ∗),Ω∗=(δ(1−δ)σ2+δ2ω∗2δω∗2δω∗2ω∗2),

$\theta^{*}=(\bar{y}\sigma^{2}+\mu \tau^{2}/n)/(\sigma^{2}+\tau^{2}/n)$, and *ω* ^∗2^ = *σ* ^2^ *τ* ^2^/(*nσ* ^2^ + *τ* ^2^).

(ii) The full conditional posterior distribution of *τ* ^2^ is the inverse-Gamma distribution
  (34) $$\begin{array}{c} \tau^{2}\;|\;\left(\theta ,D_{n}\right)\sim IG\left(c+\frac{2}{n},d+\overset{n}{\underset{i=1}{\sum }}{\left(y_{i}-\theta \right)}^{2}\right). \end{array}$$

**Proof**

(i) The unnormalized conditional density of *θ* given that *τ* ^2^ and *D*_*n*_ is proportional to

(35) $$\begin{array}{c} p\left(\theta \;|\;\tau^{2},D_{n}\right)\propto \phi \left(\bar{y};\theta ,\frac{\tau^{2}}{n}\right)\pi_{3}\left(\theta \right), \end{array}$$

where $\bar{y}=\sum_{i=1}^{n}y_{i}/n$. Thus direct application of Corollary 5 yields the result.

(ii) It is straightforward to see from (31) that the full conditional posterior distribution of *τ* ^2^ is

(36) f(τ2 ∣ θ,Dn) ∝(τ2)−(c+n/2+1)exp⁡{(d+∑i=1n(yi−θ)2)τ2},

which is a kernel of IG(*c* + 2/*n*, *d* + ∑_*i*=1_^*n*^(*y*_*i*_ − *θ*)^2^) distribution.

It is not difficult to construct the Gibbs sampling scheme working with (*θ*, *τ* ^2^) because their full conditional distributions are given in Corollary 6. A routine Gibbs sampler would work to generate posterior samples of (*θ*, *τ* ^2^). The only difficulty would lie in generating random samples from WN distribution, that is, *θ*  ∣  (*τ* ^2^, *D*_*n*_) ~ WN_(*a*,*b*)_(μ*, Ω*) as given in (32). In order to generate random samples from the WN distribution, we can make use of the stochastic representation given in (24). Also we can note that the stochastic representation (24) of the full conditional posterior distribution (32) provides the posterior mean and variance given by

(37) $$\begin{array}{c} E\left[\theta \;|\;\left(\tau^{2},D_{n}\right)\right]=\theta^{*}-\beta_{1}\kappa ,\\ \mathrm{Var}\left(\theta \;\;|\;\;\left(\tau^{2},D_{n}\right)\right)=\omega^{*2}-\beta_{2}\kappa^{2}, \end{array}$$

where *κ* = *δω* ^∗2^/*ω*_11_*, −*β*_1_, and 1 − *β*_2_ are respective mean and variance of the truncated standard normal distribution, *N*_(γ*(*a*),γ*(*b*))_(0,1) with γ*(*a*) = (*a* − *θ*_1_*)/*ω*_11_*, γ*(*b*) = (*b* − *θ*_1_*)/*ω*_11_*, *θ*_1_* = (1 − *δ*)*μ* + δθ*, and *ω*_11_^∗2^ = *δ*(1 − *δ*)*σ* ^2^ + *δ* ^2^ *ω* ^∗2^. Johnson et al. [13] shows that they are

(38) β1=ϕ(γ∗(b))−ϕ(γ∗(a))Φ(γ∗(b))−Φ(γ∗(a)), β2=γ∗(b)ϕ(γ∗(b))−γ∗(a)ϕ(γ∗(a))Φ(γ∗(b))−Φ(γ∗(a))+β12,

where *ϕ*(·) denotes the density of *N*(0,1). It is seen that each of the first terms in (37), that is, θ* and *ω* ^∗2^, is the posterior mean and variance of *θ* when the prior *π*_1_(*θ*) is put on *θ* (e.g. [15, 16]), instead of the two-stage prior of Theorem 4, that is, *π*_3_(*θ*). That is each of the second terms in the posterior mean and covariance vanishes when *θ* is assumed to be *N*(*μ*, *σ* ^2^) a priori without any interval constraint. In this sense, the second terms in (37) can be interpreted as a* constraining effect* obtained by adopting the HSGM. See, for example, Kim [3], Kim and Choi [4], and H. j. Kim and H. M. Kim [17] for practical applications of HSGM methodology to get the* constraining effect* in various constrained parameter problems.

## 5. Conclusion

This paper considered the normal models which include the two-stage prior of the normal mean, referred to as* hierarchical screened Gaussian normal model* (HSGM). The HSGM  is based on the two stages of a prior hierarch advocated by O'Hagan and Leonard [1] and elicits partial priori information obtained for the case where an interval constraint of the normal mean needs to be incorporated in the modeling but such a restriction is uncertain. Then we proposed an objective method to measure (or control) the uncertainty accounted for by HSGM. For this purpose, we derived the maximum entropy prior, reflecting the uncertainty about an interval constraint on the normal mean, by using Boltzmann's maximum entropy theorem. As a result, we found that both the maximum entropy prior and the two-stage prior in HSGM belong to the family of weighted normal distributions considered by Kim [6]. By exploring the distributional relationship between the two priors, we proposed the objective measure of uncertainty. This paper also proposed the Bayesian estimation procedure of HSGM and investigated some properties of the procedure by deriving the full conditional posterior distributions of unknown parameters under HSGM in analytic forms.
